# 965. Projecting the Potential Clinical and Economic Impact of HIV Prevention Resource Reallocation in Tennessee

**DOI:** 10.1093/ofid/ofad500.2460

**Published:** 2023-11-27

**Authors:** Ethan D Borre, Aimalohi Ahonkhai, Kyu-young Chi, Amna Osman, Krista Thayer, Anna K Person, Andrea Weddle, Clare Flanagan, April Pettit, David Closs, Mia Cotton, Allison Agwu, Michelle Cespedes, Andrea L Ciaranello, Gregg S Gonsalves, Emily P Hyle, A David Paltiel, Kenneth Freedberg, Anne M Neilan

**Affiliations:** Massachusetts General Hospital, Boston, MA; Vanderbilt University Medical Center, Nashville, TN; Massachusetts General Hospital, Boston, MA; Nashville Cares, Nashville, Tennessee; Friends for All Memphis, Memphis, Tennessee; Vanderbilt University Medical Center, Nashville, TN; IDSA, Arlington, VA; Massachusetts General Hospital, Boston, MA; Vanderbilt University Medical Center, Nashville, TN; Friends For All Memphis, Memphis, Tennessee; Friends For All Memphis, Memphis, Tennessee; Johns Hopkins University, Baltimore, MD; Icahn School of Medicine at Mount Sinai, New York, New York; Massachusetts General Hospital, Boston, MA; Yale University, New Haven, Connecticut; Massachusetts General Hospital, Boston, MA; Yale School of Public Health, New Haven, Connecticut; Massachusetts General Hospital, Harvard Medical School, Boston, Massachusetts; Massachusetts General Hospital, Boston, MA

## Abstract

**Background:**

In 2023, the State of Tennessee rejected $6.2M in US Centers for Disease Control and Prevention (CDC) HIV prevention funding, redirecting support away from men who have sex with men (MSM), transgender women (TGW), people who inject drugs (PWID), and heterosexual Black women (HSBW), and towards first responders (FR), pregnant people (PP), and survivors of sex trafficking (SST).

**Methods:**

We used a microsimulation model of HIV to compare the clinical impact of *Current*, present allocation of condoms, pre-exposure prophylaxis (PrEP), and HIV testing to CDC priority risk groups (MSM/TGW/PWID/HSBW); with *Reallocation*, diverting funds to increase HIV testing and linkage of Tennessee-determined priority populations (FR/PP/SST). We simulated Tennesseans with undiagnosed and incident HIV. Model inputs included condom use (45-67%), PrEP use (0.2-8%), HIV testing frequency (every 2.5-4.8 years), and 30-day linkage to HIV care (56-65%). We assumed *Reallocation* would reduce condom use (-4%), PrEP provision (-26%), and HIV testing (-47%) in MSM/TGW/PWID/HSBW while it would increase HIV testing among FR (+47%) and 30-day linkage to HIV care (to 100%/90%) among PP/SST; we varied these assumptions in sensitivity analyses. We conservatively assumed no impact of *Reallocation* on people with HIV already in care. 10-year model outcomes included HIV transmissions, numbers diagnosed and virologically suppressed, deaths, life-years lost, and cost/death averted.

**Results:**

Over 10 years, *Current* would lead to 6,678 total HIV transmissions, 1,989 deaths, and 85,270 life-years in Tennessee (Table 1). *Reallocation* would lead to 178 additional HIV transmissions, 219 additional HIV deaths, and 985 more life-years lost; more people would be undiagnosed and fewer would be virologically suppressed (Figure 1). Under *Current*, Tennessee would spend $263,830/death averted; under *Reallocation*, $4,133,330/death averted, a 1,470% increase in cost/death averted (Table 2). Results were most sensitive to reductions in HIV testing rates under *Reallocation.*

**Figure:**
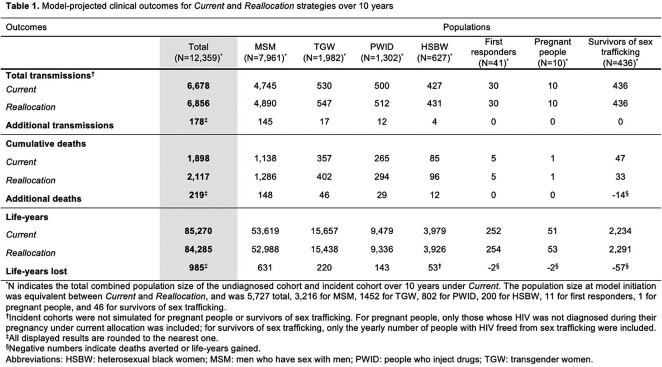

**Figure:**
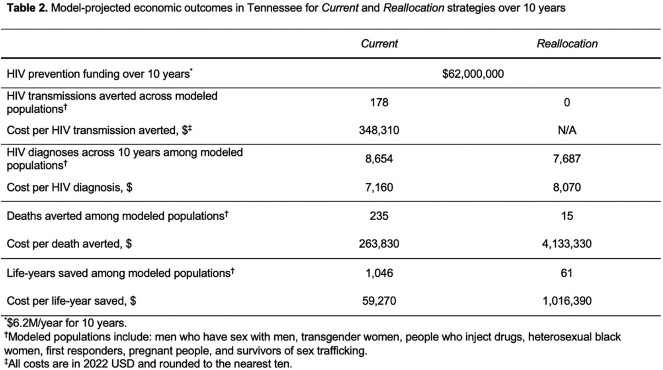

**Figure 1.**
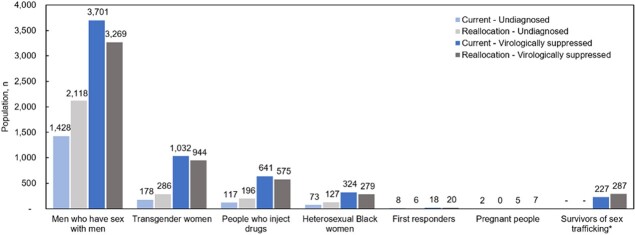
Number of simulated people with HIV in Tennessee who have undiagnosed HIV, compared to people who have diagnosed HIV and are virologically suppressed at 10 years

Figure 1 presents the projected number of people with HIV across each risk group who are undiagnosed (light shade) compared to those who are diagnosed and virologically suppressed (dark shade) at 10 years under the Current (blue) and Reallocation (gray) scenarios. Under Current, there are fewer total people with undiagnosed HIV, and more people with diagnosed HIV at year 10 compared with Reallocation. Reallocation does increase the number of people with HIV who are virologically suppressed amongst first responders, pregnant people, and survivors of sex trafficking; however, the increase is relatively small compared to the decrease in virologic suppression among men who have sex with men, transgender women, people who inject drugs, and heterosexual Black women.

*Survivors of sex trafficking are assumed to be diagnosed when freed from sex trafficking in Current and Reallocation, but in Reallocation the linkage to HIV care is assumed to increase from 56% to 90%.

**Conclusion:**

Reallocation of HIV prevention funding in Tennessee would greatly harm MSM/TGW/PWID/HSBW, confer minimal benefits for FR/PP/SST, and cannot be justified on either clinical or epidemiological grounds.

**Disclosures:**

**Allison Agwu, MD, MSc**, Gilead: Board Member|Gilead: Grant/Research Support|Merck: Advisor/Consultant|Merck: Grant/Research Support|ViiV: Board Member

